# Multiplex attacks in sigmoid volvulus

**DOI:** 10.12669/pjms.40.6.9172

**Published:** 2024-07

**Authors:** Esra Disci, Rifat Peksoz, Enes Agirman, Sabri Selcuk Atamanalp

**Affiliations:** 1 Esra Disci, MD. Associate Professor, Department of General Surgery, Faculty of Medicine, Ataturk University, Erzurum, Turkey; 2 Rifat Peksoz, MD. Assistant Professor, Department of General Surgery, Faculty of Medicine, Ataturk University, Erzurum, Turkey; 3 Enes Agirman, MD. Assistant Professor, Department of General Surgery, Erzurum City Hospital, Erzurum, Turkey; 4 Sabri Selcuk Atamanalp, MD. Professor, Department of General Surgery, Faculty of Medicine, Ataturk University, Erzurum, Turkey

**Keywords:** Sigmoid volvulus, Multiple attacks, Multiplex attacks

## Abstract

**Objectives:**

Sigmoid volvulus (SV) recurs in about one quarter of the patients, whereas multiplex (≥3) attacks are quite rare and attacks with five or more times are extremely rare. The aim of this study was to evaluate multiplex SV attacks in our series and worldwide data.

**Methods:**

In Ataturk University Faculty of Medicine Department of General Surgery, among 1,071-case SV series, data were evaluated retrospectively in 612 patients, while prospectively in 459 with respect to age, gender, previous volvulus attacks, and prognosis. Worldwide data were obtained from Web of Science database and they were compared with our results.

**Results:**

Mean SV attack count, multiple- (≥2) and multiplex- (≥3) attack rates were 1.4, 26.1%, and 4.2%, respectively, in our series, while they were 1.7, 26.7%, and 3.2%, respectively, in worldwide data (p>0.05, in all). In our series, recurrence rates were 26.1%, 19.3%, and 51.2%, respectively, (p<0.001, in all), while mortality rates were 7.3%, 13.7%, and 19.5%, respectively, (p<0.001, in all) in single-, double-, and multiplex- (≥3) attack patients.

**Conclusion:**

Although multiplex (≥3) attacks are uncommon in SV, when it goes up, elective surgery must be considered in selected cases to avoid repetitive attacks and related high mortality.

## INTRODUCTION

Sigmoid volvulus (SV) tends to recur particularly in patients treated with nonoperative or operative decompression alone.^1^ In SV, multiple (≥2) attacks are reported to be between 4.0%-88.9% in the literature.^2^,^3^ However, multiplex (≥3) attacks are quite rare and attacks more than five times are extremely rare.^3^-^7^ According to a search of the last 56-years’ literature (between 1967 and 2023) in Web of Science^8^ database, the number of the patients with five or more SV attacks can be counted on the fingers of one hand. In relation to the causes and results of this unfamiliar clinical situation, we want to share our 1,071-patient SV experience, the most detailed monocenter SV series worldwide including a case with 25 attacks, which constitutes the highest SV attack count reported to date.^1^,^9^

## METHODS

In Ataturk University Faculty of Medicine Department of General Surgery, among total 1,071 SV patients, a retrospective examination was performed in the medical records of 612 cases (57.1%, from June 1966 to June 1986), whereas the data of 459 patients (42.9%, from June 1986 to July 2023) were evaluated prospectively. The age, gender, previous volvulus attacks, and prognosis of each case were recorded. In routine practice, endoscopic decompression was preferred as first-line management, whereas patients with acute abdominal findings or bowel gangrene evidences, or those with unsuccessful nonoperative decompression required emergency surgery. In selected successfully decompressed cases, particularly in those with recurrent attacks, elective surgery was suggested and applied in the admitters, while the others were discharged.

As worldwide data, publications related to SV were investigated through Web of Science^8^ database under the head of ‘sigmoid volvulus’. Among 1,199 papers, those with undetailed data were excluded and 532 papers were evaluated to compare the results of multiple (≥2) and multiplex (≥3) SV attacks with ours.

SPSS v22.0 program (IBM Corporation, Armonk, New York, United States) was used in statistical analysis. Data were expressed as numerical variables or percentages for categories. Categorical variables were compared by using Chi-Square, Linear-by-Linear association, Fisher exact, and Fisher freeman Halton tests. Numerical variables for more than two groups were compared by using ANOVA. The significance level was set up as p<0.05.

### Ethical Approval:

Informed consent was obtained from all patients or executors, and this study was performed by ethical permission of the institutional review board (Ethical Committee of Ataturk University Faculty of Medicine, 88/27.01.2022).

## RESULTS

The worldwide data obtained from Web of Science^8^ database including our series on multiple (≥2) and multiplex (≥3) SV attacks are demonstrated in Table-I, the results of our study together with the worldwide data including statistical analyses are given in Table-II, and the detailed results of our study and related statistical analyses are shown in Table-III.

**Table-I:** Multiple (≥2) and multiplex (≥3) sigmoid volvulus attacks in worldwide literature and in our series.

Author	Year	Case	Attack						

			Multiple (%)	2	3-5	6-10	≥11	Maximal	Mean
String and DeCosse^2^	1971	25	1 (4.0)						
Arnold and Nance^10^	1973	99	30 (30.3%)						1.4
Ryan^11^	1982	66	18 (27.3)						
Arigbabu et al.^12^	1985	92	66 (71.7)						
Brothers et al.^13^	1987	39	5 (12.8)						
Oncu et al.^14^	1991	17	8 (47.1)						
Asbun et al.^15^	1992	230	16 (7.0)						
Grossmann et al.^16^	2000	228	30 (13.2)						
Bhatnagar et al.^17^	2004	76	16 (21.1)						
Cartwright-Terry et al.^3^	2007	9	8 (88.9)	4	4				2.4
Heis et al.^4^	2008	32	13 (40.6)	10	3				1.5
Larkin et al.^18^	2009	27	16 (59.3)						
Jangjoo et al.^5^	2010	75	17 (22.7)		1			5	
Gupta et al.^19^	2011	72	11 (15.3)						
Swenson et al.^20^	2012	50	19 (38.0)						
Maddah et al.^21^	2014	217	28 (12.9)						
Bruzzi et al.^22^	2015	65	25 (38.5)						
Johansson et al.^6^	2018	168	108 (64.3)				1	16	1.8
Heo et al.^23^	2019	51	8 (15.7)						
Gonzales-Urquijo et al.^24^	2020	34	4 (11.7)						
Total		1.672	447 (26.7)	14	8	0	1	16	1.7
Present series	2023	970	253 (26.1)	212	35	3	3	25	1.4

**Table-II:** The results of our study together with the worldwide data on multiple (≥2) and multiplex (≥3) sigmoid volvulus attacks and related statistical analyses.

Attack	Our series	Worldwide series	Statistical analysis
Mean	1.4 (1,370/970)	1.7 (511/308)	Chi-square test p>0.05
Multiple (≥2)	26.1% (253/970)	26.7% (447/1,672)	Chi-square test p>0.05
Multiplex (≥3)	4.2% (41/970)	3.2% (9/284)	Chi-square test p>0.05

**Table-III:** The detailed results of our study on multiple (≥2) and multiplex (≥3) sigmoid volvulus attacks and related statistical analyses.

Attack	Single-attack recurrence	Double-attack recurrence	Multiplex- (≥3) attack recurrence	Statistical analysis
Age, range	57.9 years (7-98)	63.3 years (17-89)	68.6 years (23-91)	Post-Hoc test p>0.05, double-multiplex p<0.001, others
Male/Female	4.2 (579/138)	5.8 (181/31)	7.2 (36/5)	Chi-square test p>0.05
Recurrence	26.1% (253/970)	19.3% (41/212)	51.2% (21/41)	Chi-square test p<0.001
Mortality	7.3% (52/717)	13.7% (29/212)	19.5% (8/41)	Chi-square test p<0.001

In our series, the mean attack count was 1.4 in total (range: 1-25 attacks), while it was 2.6 in recurrent cases. Multiple (≥2) attacks were determined in 253 patients (26.1%), while multiplex (≥3) attacks were seen in 41 (4.2%) of our 970 cases, in whom medical history was obtained. In 212 (83.8%) of recurrent cases, the attacks count was two, while it was 3-5 in 35 (13.8%), 6-10 in 3 (1.2%), and ≥11 in 3 (1.2%). Regarding the worldwide data, the mean attack count was reported to be between 1.4-2.4 (mean 1.7 attacks), while multiple- (≥2) attack rate was determined as 26.7% (447/1,672) and multiplex- (≥3) attack rate was found as 3.2% (9/284) in accessible literature data. In statistical evaluation, the mean SV attack count of our series was similar to that of the worldwide data (1.4 vs. 1.7, p>0.05). Likewise, our multiple- (≥2) attack (26.1% vs. 26.7%, p>0.05) and multiplex- (≥3) attack incidences (4.2% vs. 3.2%, p>0.05) were not statistically different when compared with that of the worldwide series.

Detailed analyses of our series demonstrated that, the mean age was 57.9 years (range: 7-98 years) in single-attack recurrent patients, while it was 63.3 years (range: 17-89 years) in double-attack recurrent patients, and 68.6 years (range: 23-91 years) in multiplex- (≥3) attack recurrent cases, and the differences were statistically significant except for the comparison of double- and multiplex- attack groups (p<0.001, p<0.001, and p>0.05, respectively). Unsurprisingly, male/female ratios were 4.2, 5.8, and 7.2, respectively, in single-, double- and, multiplex- (≥3) attack patients, but the differences were not statistically significant (p>0.05, in all). Conversely, recurrence was statistically more common in cases with multiplex (≥3) attacks when compared with that of single- and double-attacks patients (51.2% vs. 26.1% and 19.3%, p<0.001, in all). Similarly, the mortality rate was statistically higher in recurrent cases, particularly in those with multiple (≥3) attacks (19.5% vs. 7.3% and 13.7%, p<0.001, in all).

## DISCUSSION

Despite a relatively low mean attack count of 1.4 in our series, due to the relatively high predisposition to recurrence in SV, the mean attack count is reported to be between 1.4-2.4 (mean 1.7 times) in the worldwide literature.^2^-^6^,^10^-^24^ Multiple (≥2) attacks are relatively high in SV, which is 26.1% in our series, while 4.0%-88.9% (mean 26.7%) over the world.^2^-^6^,^10^-^24^ However, multiplex (≥3) attacks are quite rare (4.2% in our series and mean 3.2% in worldwide data) and attacks ≥5 times are extremely rare, which are limited to a few cases.^3^-^7^ When the highest number of attack is considered in the worldwide literature, our patient with 25 attacks breaks the record, while Johansson et al.^6^ rank number two with their 16-attack case, and we rank number tree by way of our another patient with 14 attacks. Regarding the cause of such high recurrence counts of our two cases, their poor general states of health, American Society of Anesthesiologists (ASA) four scores arising from serious comorbidities, were an impediment to a planned surgery in addition to their refuse a simpler procedure such as percutaneous endoscopic colopexy.

The primary factor in the relatively high multiple- (≥2) SV attack incidence in our region (26.1%) and over the world (26.7%) is the reluctance of most patients to elective surgery following endoscopic decompression.^1^ Elective sigmoid colectomy is the most important recurrence-preventive action in SV.^2^,^3^,^5^,^6^,^9^,^18^,^20^-^22^ However, in our experience, most discharged patients fail to return elective surgery due to the stolidity arising from successful endoscopic decompression, which incapableness may be prevented by performing elective surgery in the first admission and during 1-5 days following decompression.^9^,^25^ Another important expositive factor in the high SV recurrence rate is the dilemma of the decision making process in elective surgery.

The intended patient population for elective surgery is described as ‘well-conditioned and nonelderly cases’ in the literature,^3^,^5^,^6^,^10^,^20^-^22^ which description is relatively far from objectivity. However, in our opinion, the usage of an objective criterion such as ‘the patients with ASA score 1-3 and aged <70-75 years are presumptive candidates for elective surgery’ is more practical.^25^ Lastly, the difficulty in healthcare access consisting of elective surgery is another nontrivial factor in the high SV recurrence and related heavy prognosis in some developing or underdeveloped countries.^4^,^12^,^16^

The present study demonstrated that, multiplex (≥3) SV attacks are quite rare in our region (4.2%) and over the world (3.2%). Additionally, attacks with five or more times are limited to a few cases. Most likely due to rarity of this clinical situation, both its causes and results are not clearly idntified.^8^ In our opinion and experience, the major cause of the rarity of the multiplex attacks is the treatment of most patients with surgical sigmoid colectomy following repetitive attacks. Moreover, death of some cases due to the major SV complications reduces the occurrence of multiplex attacks. Regarding the clinical results of the multiplex (≥3) attacks in SV, although every attack carries in a certain extent of mortality and morbidity risk, in our experience, this tough situation carries some unexpected advantageous outcomes. First, such patients are familiar to the clinical features of SV and they generally come to the hospital without delay. Second, such cases diagnose SV by themselves, which typically causes early diagnosis and treatment. Third, although the repetitive attacks frequently make the recurrent torsion easier, they also expedite the detorsion due to anatomical predisposition, which may result in spontaneous decompression.^7^

In the evaluation of the age and genders, similar to primary SV, double and multiplex (≥3) attacks were more common in elderly men in our SV series, while they were not present in childhood, which may be explained by the high incidence of dolichosigmoid (the presence of an elongated and dilated sigmoid colon with a long mesentery) in males and elderly individuals.^1^ Likewise, the high recurrence rate in our patients with multiplex (≥3) attacks when compared with that of single-attack recurrent patients and double-attack recurrent cases (51.2% vs. 26.1% and 19.3%) demonstrated that the recurrence risk increasingly continued following the third and next attacks. In our opinion and experience, the relatively low recurrence rate following the second attack may be explained by the treatment of some patients with elective surgery following the second attack, while the causes of the relatively high recurrence rate following the third and next attacks may be the rarity of the cases eligible for the elective surgery in the remained group as well as the anatomical predisposition to the recurrence arising from the repetitive attacks. If this theory is true, the management of the suitable patients with elective surgery following the first or at least second attack may be the optimal choice in SV. On the other hand, the high mortality rate in recurrent cases, particularly in those with multiple (≥3) attacks (19.5% vs. 7.3% and 13.7%) in our series obligates us as well as all the practitioners to use the elective surgery as the preferred treatment option without any delay.

Finally, the decrease in the SV incidence of our prospective series (459 patients in 37 years, 12.4 cases per year) when compared with our retrospective series (612 patients in 20 years, 30.6 cases per year) may principally be ascribed to the westernization of dietary habits, which is a preventive factor in the development of dolichosigmoid, an anatomical predisposition for both primary and recurrent SV.^1^,^4^

### Limitations:

It includes the rarity of the multiplex SV attacks in our region and over the world in addition to the partial retrospective consideration of our data are the conspicuous limitations of the present work. However, more extensive studies require many decades to obtain more practical results on this extremely rare clinical entity.

## CONCLUSIONS

Recurrence occurs in about one fourth of SV patients, whereas multiplex (≥3) attacks are uncommon. In our experience, most discharged patients fail to return for elective surgery. For this reason, elective surgery in the first admission and during 1-5 days following decompression may be the optimal choice. An important expositive factor in the high SV recurrence rate is the dilemma of the decision making process in elective surgery. In our opinion, ASA score 1-3 and aged <70-75 years are presumptive candidates for elective surgery. Due to the poor prognosis of the observation without sigmoid colectomy in cases with repetitive attacks, elective surgery following the first or at least second attack is recommended.
